# Effects of an Experimental Water-level Drawdown on Methane Emissions from a Eutrophic Reservoir

**DOI:** 10.1007/s10021-017-0176-2

**Published:** 2017-09-05

**Authors:** Jake J. Beaulieu, David A. Balz, M. Keith Birchfield, John A. Harrison, Christopher T. Nietch, Michelle C. Platz, William C. Squier, Sarah Waldo, John T. Walker, Karen M. White, Jade L. Young

**Affiliations:** 1 National Risk Management Research Laboratory, United States Environmental Protection Agency, Office of Research and Development, 26 W Martin Luther King Dr, Cincinnati, Ohio 45268 USA; 2 Pegasus Technical Services, Cincinnati, Ohio USA; 3 School of the Environment, Washington State University, Vancouver, Washington USA; 4 National Enforcement Investigations Center, United States Environmental Protection Agency, Denver, Colorado USA; 5 National Risk Management Research Laboratory, United States Environmental Protection Agency, Office of Research and Development, Durham, North Carolina USA; 6 United States Army Corps of Engineers, Louisville, Kentucky USA

**Keywords:** methanogenesis, biogeochemistry, carbon, anthropogenic, management, aquatic

## Abstract

Reservoirs are a globally significant source of methane (CH_4_) to the atmosphere. However, emission rate estimates may be biased low due to inadequate monitoring during brief periods of elevated emission rates (that is, hot moments). Here we investigate CH_4_ bubbling (that is, ebullition) during periods of falling water levels in a eutrophic reservoir in the Midwestern USA. We hypothesized that periods of water-level decline trigger the release of CH_4_-rich bubbles from the sediments and that these emissions constitute a substantial fraction of the annual CH_4_ flux. We explored this hypothesis by monitoring CH_4_ ebullition in a eutrophic reservoir over a 7-month period, which included an experimental water-level drawdown. We found that the ebullitive CH_4_ flux rate was among the highest ever reported for a reservoir (mean = 32.3 mg CH_4_ m^−2^ h^−1^). The already high ebullitive flux rates increased by factors of 1.4–77 across the nine monitoring sites during the 24-h experimental water-level drawdown, but these emissions constituted only 3% of the CH_4_ flux during the 7-month monitoring period due to the naturally high ebullitive CH_4_ flux rates that persist throughout the warm weather season. Although drawdown emissions were found to be a minor component of annual CH_4_ emissions in this reservoir, our findings demonstrate a link between water-level change and CH_4_ ebullition, suggesting that CH_4_ emissions may be mitigated through water-level management in some reservoirs.

**Electronic supplementary material:**

The online version of this article (doi:10.1007/s10021-017-0176-2) contains supplementary material, which is available to authorized users.

## Introduction

Methane (CH_4_) is a potent greenhouse gas and the second largest contributor to climate change (IPCC 2013). Reservoirs, which are created when landscapes are flooded behind dams, are a globally significant source of CH_4_ to the atmosphere (Barros and others 2011; Bastviken and others 2011; Deemer and others 2016). Reservoirs are extremely numerous, covering more than 30 million ha of the earth’s land surface (Downing and others 2006), and the global surface area of reservoirs is expected to increase substantially over the coming decades as developing countries turn to hydropower to meet growing energy demands (Zarfl and others 2015). Methane is created in reservoirs through methanogenesis, a type of microbial metabolism that yields energy from the degradation of organic matter in low oxygen environments. Reservoir sediments are often enriched with organic matter derived from river inflows and internal algal production, among other sources. Relative to lakes, organic matter loading to reservoirs from river inflows can be particularly high due to their characteristically large ratio of catchment area to lake area (Kimmel and others 1990; Knoll and others 2014; Hayes and others 2017). Furthermore, the bottom waters of reservoirs are often anoxic during periods of thermal stratification, providing the mix of high organic matter and low oxygen conditions conducive to CH_4_ production.

Efforts to use published data to estimate regional/global scale CH_4_ emissions from reservoirs, or to identify large-scale factors driving emission rates (that is, latitude, reservoir age), have been complicated by uncertainty in emission rate measurements (Barros and others 2011; Bastviken and others 2011; Deemer and others 2016). An important source of uncertainty is the degree to which individual studies address high emission rates associated with spatial hot spots and temporal hot moments (McClain and others 2003; Schilder and others 2013; Schilder and others 2016; Wik and others 2016). Methane emission rates can exhibit extreme intra-reservoir spatial heterogeneity. For example, river–reservoir transition zones can be CH_4_ emission hot spots due partly to high sedimentation rates which promote the delivery of organic matter to anoxic sediments (DelSontro and others 2011; Beaulieu and others 2014; Beaulieu and others 2016). Grinham and others (2011) reported that 97% of the CH_4_ emitted from the surface of a subtropical reservoir was derived from approximately 5% of the reservoir surface area located immediately downstream of the main tributary input. Similarly, low water depths, which promote the transfer of CH_4_-rich bubbles from the sediment to the atmosphere, and organic-rich sediments in littoral zones, can also lead to emission hot spots in reservoirs (Juutinen and others 2003; Hofmann 2013). Methane emission rates in reservoirs can also exhibit hot moments where elevated rates occur for brief periods. For example, CH_4_ that accumulates under ice in winter, or in the hypolimnion during periods of thermal stratification, can vent to the atmosphere during short windows following spring ice melt and fall turnover, respectively (Michmerhuizen and others 1996; Schubert and others 2012). These lake mixing patterns give rise to brief periods of intense emissions that have been shown to account for up to 45% of annual emissions (Michmerhuizen and others 1996; Bastviken and others 2004). Improving our understanding of spatiotemporal patterns in reservoir-CH_4_ emissions is critical to including these systems in greenhouse gas (GHG) inventories (IPCC 2006; Fearnside 2015) and assessing the GHG footprint of hydropower (Hertwich 2013; Scherer and Pfister 2016).

Another process which may give rise to periods of elevated CH_4_ emission rates in reservoirs is water-level drawdown. Water-level drawdowns are frequently conducted in reservoirs to increase flood storage capacity, generate hydropower, or to perform dam maintenance, among other reasons (Harrison and others 2017; Hayes and others 2017). Changes in water levels translate to shifts in hydrostatic pressure, which can play an important role in regulating CH_4_ emissions. Here we treat hydrostatic pressure as the pressure at the sediment–water interface due to the weight of the overlying column of water and the atmosphere. Under conditions of constant hydrostatic pressure, gases produced via microbial activity dissolve in sediment porewaters until the combined partial pressures of the dissolved gases exceed the hydrostatic pressure, at which point bubbles are formed (Chanton and Whiting 1995). A fraction of these bubbles will grow large enough for their buoyancy to overcome the combined hydrostatic pressure and cohesive strength of the sediment, at which point they will migrate upward through the sediment. Rates of gas production via microbial activity often exceed rates of gas loss via ebullition during warm weather months, however, leading to the accumulation of large volumes of gas in reservoir sediment (Martinez and Anderson 2013; Tyroller and others 2016). When water levels fall, the hydrostatic pressure holding the bubbles in place diminishes, potentially leading to the release of a large fraction of the stored CH_4_ to the atmosphere. This phenomenon is well documented in marine systems where tide-driven fluctuations in water levels can trigger the release of CH_4_ bubbles from coastal marsh sediments (Chanton and others 1989) and seafloor CH_4_ seeps (Boles and others 2001).

Water-level declines have also been shown to trigger ebullition in reservoirs (Eugster and others 2011; Deshmukh and others 2014; Harrison and others 2017). Harrison and others (2017) recently demonstrated that ebullition during drawdown events can constitute more than 90% of annual CH_4_ emissions from reservoirs in the Pacific Northwest (PNW) of the USA, suggesting that monitoring emissions during periods of water-level decline may be critical to constructing accurate annual CH_4_ budgets.

In this research, we investigated spatial and temporal patterns in CH_4_ emission rates from Harsha Lake, a reservoir draining an agricultural watershed in the Midwestern USA. Like the PNW reservoirs, water levels in Harsha Lake are drawn down in the fall to increase winter flood storage capacity. Unlike the PNW reservoirs, however, Harsha Lake supports a very high ebullitive CH_4_ flux during the summer months (Beaulieu and others 2014, 2016), when the reservoir is managed for constant water levels. It was unclear whether the already high ebullition rate would be further enhanced by falling water levels in the fall.

We instituted an ecosystem-scale experiment by lowering water levels 27 times faster than normal for the first 24 h of the 3-month drawdown and then reducing the drawdown rate to that prescribed by the management schedule for the balance of the drawdown. Although the experimental drawdown rate (0.46 m day^−1^) was much higher than that prescribed by the management schedule, it is realistic for this and other reservoirs. For example, the experimental drawdown rate is similar to drawdown rates that occur when flood waters are released from the reservoir following large storms and is comparable to drawdown rates reported for seven PNW reservoirs (0.12–0.66 m day^−1^) (Harrison and others 2017). We monitored ebullition at nine sites spanning the length of the reservoir allowing us to determine (1) whether ebullition rates are related to rate of water-level decline and (2) whether the ebullitive response to the drawdown varied across the reservoir (that is, shallow vs deep waters).

## Methods

### Site Description

**Figure 1:**
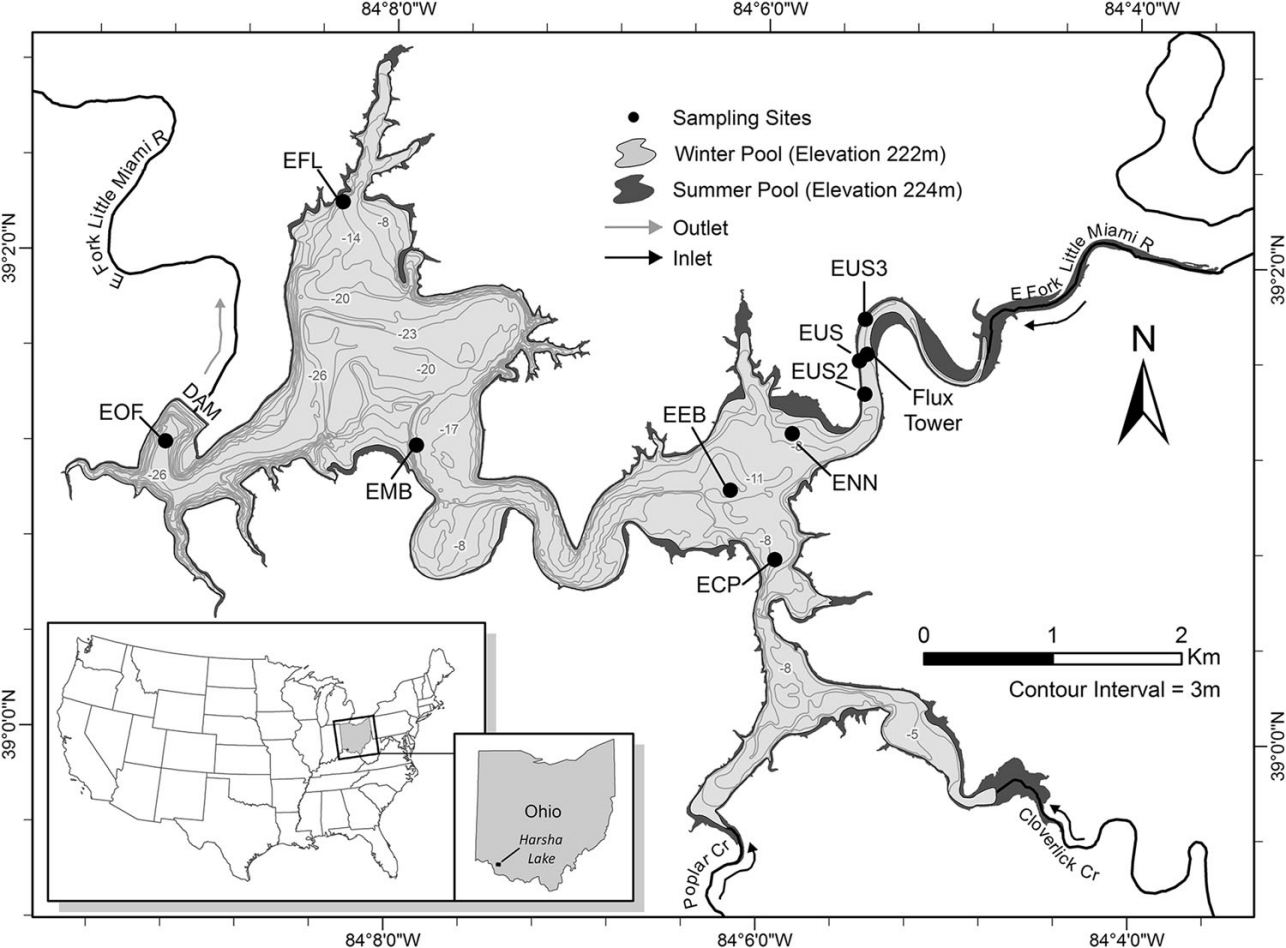
William H. Harsha Lake (Harsha Lake), tributaries, outlet, and sampling sites.

William H. Harsha Lake is a 7.9-km^2^ eutrophic reservoir draining an 882 km^2^ watershed in Ohio, USA (Figure 1). The reservoir was constructed in 1978, has a water storage capacity of 1.1 × 10^8^ m^3^, a maximum depth of 32.8 m during the summer months, and is monomictic. Large portions of the watershed are managed for agricultural production (63% cultivated crops and pasture), and the reservoir is frequently on the state’s advisory list for recreational contact due to the abundance of harmful blue-green algae (http://epa.ohio.gov/habalgae.aspx).

The water-level management plan for the reservoir, known as the guide curve, targets a pool elevation of 223.4 m above mean sea level (m-msl) from May 1 through September 1 (that is, summer pool), at which point the pool is lowered at a constant rate to 221.9 m-msl by Dec. 1 (that is, winter pool), for a total drawdown of 1.5 m over a 3-month period. This period of water-level decline is commonly referred to as the ‘fall drawdown.’ Winter pool is maintained until April 1, at which point the water level is raised at a constant rate to summer pool by May 1. Low water levels are maintained during the winter, in part, to enhance flood protection.

To test the hypothesis that ebullition rates are dependent on the rate of water-level decline, we imposed an experimental treatment on the guide curve. We delayed the fall drawdown until 9:00 am on September 14, 2015, and then dropped the water level by 0.46 m over 24 h, a drawdown rate 27-fold greater than dictated by the guide curve. After the experimental drawdown, the rate of water-level decline was reduced to that prescribed by the guide curve.

### Sampling Strategy

Nine monitoring sites were established across the reservoir (Figure 1). Six of the sites (EOF, EMB, EFL, EEB, ECP, and ENN) were collocated with long-term water quality monitoring sites sampled by the US Environmental Protection Agency and the US Army Corps of Engineers. Two additional sites (EUS2 and EUS3) were located along the river–reservoir transition zone, a known ebullition hot spot (Beaulieu and others 2014, 2016). The ninth site (EEB) was added to better characterize the strong east to west gradient in CH_4_ emissions previously documented for the eastern basin.

#### Routine Sampling Campaign

All sites were sampled every other week from May 1 to July 6 and weekly from July 15, 2015, to December 11, 2015. Continuously recording gas traps (for example, automated traps, see ‘Ebullition rate measurement’ below) were used to measure volumetric ebullition rates, and discrete gas samples were collected from the traps for analysis of gas composition. Dissolved gas samples were collected from near the air–water interface and used to estimate diffusive CH_4_ emission.

#### Drawdown Measurement Campaign

Ebullition rates were measured during the week of the drawdown experiment (September 10–15, 2015) using a version of the gas trap that accommodated higher ebullition rates, but was not equipped for continuous data logging (that is, passive traps, see ‘Ebullition rate measurement’ below). As with the routine sampling campaign, gas samples were collected from the traps for composition analysis. Emission rates were also measured using an eddy covariance system beginning 2 days before and ending 2 days after the drawdown experiment (September 12–17, 2015). Although diffusive CH_4_ emissions were included in the eddy covariance-based estimates of CH_4_ flux, dissolved gas concentrations and diffusive emissions were not directly monitored during the drawdown experiment.

### Ebullition Rate Measurement

We measured ebullition rates using automated gas traps, with the exception of the week of the drawdown experiment (September 10–15, 2015) when we used passive gas traps. The design of the automated traps is described in Varadharajan and others (2010). Briefly, the automated trap consists of an inverted funnel (0.56 m diameter) suspended from a buoy and connected to a 0.97 m long by 1.25- or 2.5-cm-diameter vertical pipe that serves as a collection chamber. Accumulating gas caused the pressure in the collection chamber to increase, which was recorded every 5 min using a differential pressure sensor (Honeywell) and datalogger (U12–013, Onset Systems, Massachusetts, USA). The automated trap provides continuous measurements of gas volume until the signal from the differential pressure sensor reaches a maximum value, which occurs at a gas column height of 53–80 cm, depending on differences in the condition of individual sensor circuits. The sensors reached their maximum values in hours to days during this study (see Results below). The 1.25 and 2.5 cm collection chambers held up to 250 and 600 ml of gas, respectively, before overflowing into the funnel.

The relationship between the voltage readings from the differential pressure sensor and the height of the gas column in the collection chamber was established for each sensor circuit using laboratory calibrations performed at the beginning and end of the monitoring period. The height of the gas column in the trap (*h*_g_; m) is calculated as:

1 $$ h_{\text{g}} = m(V_{\text{out}} - V_{\text{Zero}} )  $$

where *m* = sensor-specific laboratory calibration (m V^−1^), *V*_out_ = voltage output from the differential pressure sensor, and *V*_zero_ = voltage output from the differential pressure sensor when there is no gas in the trap immediately following deployment. The volume of gas (*V*_g_; m^3^) in the traps was calculated by multiplying *h*_g_ by the cross sectional area of the collection chamber.

During the drawdown experiment, we replaced the gas collection chamber and electronics on the automated traps with a 5-m length of 5.7-cm-diameter tubing. These ‘passive’ traps, which were not equipped to automatically record gas volumes, were designed to accommodate the high ebullition rates anticipated during the experimental drawdown. The anticipated ebullition rates would have quickly filled the smaller gas collection chambers on the automated traps, thereby greatly reducing the maximum deployment duration. Although we did not conduct a direct comparison of passive and automated gas traps, the systems only differed in the dimensions of the collection chamber (that is, identical funnels, buoys, weights, rope, anchor, etc.), which is unlikely to have affected the measured ebullition rates. The top of the collection chambers on both automated and passive traps was equipped with a rubber septum through which the accumulated gas was sampled with a syringe and needle.

Both automated and passive traps were deployed approximately 0.5 m below an anchored buoy. At each sampling visit, the total volume of gas in the automated or passive trap was measured and up to three 20 ml gas samples were collected in pre-evacuated 12-ml screw-top soda glass vials capped with a silicone-coated Teflon septa stacked on top of a chlorobutyl septa (Labco Ltd., UK). Gas samples were analyzed for CH_4_ concentration using a gas chromatograph equipped with an autosampler, 250 µl sample loop, and flame ionization detector (Bruker 450 GC, USA). Mean variability among triplicate field samples was 1.9%.

Ebullitive CH_4_ flux (mg CH_4_ m^−2^ h^−1^) is calculated as:

2 $$ {\text{Ebullitive}}\;{\text{CH}}_{4} \;{\text{flux}} = \frac{{V_{\text{g}}  [{\text{CH}}_{4} ]}}{{\left( {T_{\text{F}} - T_{\text{I}} } \right)A_{\text{F}} }} $$

where *V*_g_ is the volume of gas in the trap (l), [CH_4_] is the CH_4_ concentration in the gas (mg CH_4_ l^−1^), *T*_I_ is the time the trap was deployed, and *A*_F_ is the cross-sectional area of the funnel (m^2^). For passive traps, *T*_F_ is the time the trap was sampled at the end of a deployment. For automated traps, *T*_F_ is either the time the pressure sensor maxed out, or the time the trap was sampled, depending on whether or not the pressure sensor was saturated during the deployment. The calculated ebullition rate therefore represents an integration of the total gas captured between *T*_I_ and *T*_F_. The 5-min logging interval was used to accurately determine the date and time that the pressure sensor reached it maximum value (*T*_F_), rather than to calculate ebullition rates at a 5-min frequency.

Root-sum-squared error propagation was used to estimate uncertainty in ebullitive CH_4_ flux (equation 2) based on uncertainty in *V*_g_. For the passive traps, *V*_g_ was directly measured using syringes and uncertainty was determined by the precision of the syringes, which was 0.5 and 1.0 ml for the 30- and 140-ml syringes, respectively. Uncertainty in *V*_g_ for the automated traps was calculated by propagating error in m and electronic noise through equation 1 as described in Varadharajan and others (2010). The automated trap *V*_g_ error term also includes a 2-ml dead volume error, which accounts for gas that could be trapped in the fittings at the top of the collection chamber. Error in the other terms in equation 2 was negligible in comparison with the error in *V*_g_.

We used control traps to assess the effect of gas exchange across the air–water interface in the collection chamber on the composition of gas stored in the traps. Control traps consisted of an automated trap (see above) with a 1 m × 1 m plexiglass sheet suspended below the funnel to prevent bubbles from entering the collection chamber. On five separate occasions, we added a mix of 90% CH_4_ and 10% CO_2_ to the control traps at the beginning of a 5- to 9-day deployment. The mean rate of change of the CH_4_ partial pressure in the control traps (−0.28% day^−1^) during the deployments was not significantly different than 0 (one-sided *t* test, *p* = 0.15), indicating that the CH_4_ content of trapped bubbles was stable for at least 9 days. Harrison and others (2017) report that the CH_4_ content of stored gas remained constant for 55 days in similar traps.

Data from deployments where the pressure sensor had not yet reached the maximum value at the time of sampling were used to compare the volume estimates derived from the sensor to those measured manually. The sensor-derived volume estimates were well correlated with manual measurements (*r* ^2^ = 0.98), and the slope of the regression between the two volume estimates was not statistically different than 1 (0.96 ± 0.059) (Figure S1), indicating that the sensor-derived estimates are precise and accurate.

### Eddy Covariance Tower

Eddy covariance (EC) measurements of CH_4_ fluxes from the river–reservoir transition zone (39°01′37.08″N, 84°05′26.82″W; Figure 1) started on September 12, 2015, at 8:30 pm, approximately 36 h before the experimental drawdown began and continued through September 17, 2015, at 4:30 pm, for a total of 232 30-min flux measurement periods. The EC system was mounted at 1.22 m above the pre-drawdown water surface by attaching the instrumentation to a large stable tree stump that protruded ~2 m above the water surface. An ultrasonic anemometer was used to measure 3-dimensional wind speed and direction (Model 81000, R. M. Young Company, Traverse City, MI, USA), and an LI-7700 open-path gas analyzer was used for fast measurements of CH_4_ partial pressure (LI-COR Inc., Lincoln, NE, USA). Carbon dioxide (CO_2_) and H_2_O partial pressures were measured with an open-path infrared gas analyzer (LI7500A, LI-COR Inc., Lincoln, NE, USA), and the flux data were logged at 10 Hz using the LI7550 control unit (LI-COR Inc., Lincoln, NE, USA). The gas analyzers were mounted on either side of the ultrasonic anemometer so that the center of the optical paths was at the same height as the center of the ultrasonic anemometer (that is, vertical separation of zero). The horizontal distance between the ultrasonic anemometer and each gas analyzer was 30 cm. The system was powered with solar panels and 12 V batteries.

### Data Processing

Fluxes of CH_4_, CO_2_, sensible heat and latent heat were calculated from the 10 Hz data using the software package EddyPro^®^ (Version 6.1.0, LI-COR Inc., Lincoln, NE) and a flux-averaging interval of 30 min. An optimal averaging interval is as short as possible to characterize temporal patterns in fluxes, but long enough to measure flux contributions from low-frequency eddies. Previous work has shown that low-frequency contributions to turbulent fluxes typically converge at 30 min. Absolute limits were applied to the raw data, which were also de-spiked (Vickers and Mahrt 1997) and de-trended using block averaging. Time lags were detected and compensated for via covariance maximization. The Webb, Pearman, Leuning (WPL) correction for density effects was applied for both CH_4_ and CO_2_ fluxes (Webb and others 1980), and the McDermitt and others (2011) spectroscopic correction for absorption line broadening due to temperature, pressure, and water vapor effects was applied to the LI-7700 CH_4_ measurements. Spectral corrections for high- and low-pass filtering were applied (Moncrieff and others 1997, 2004), and flux footprints were estimated using the method of Kormann and Meixner (2001) and Kljun and others (2004). The flux calculations took into account the increase in the sensor height above the water surface as the lake level was drawn down using a dynamic metadata file in EddyPro^®^.

Several quality assurance criteria were applied to the 30-min CH_4_ fluxes. Time periods that did not meet the requirements for stationarity and developed turbulent conditions were filtered (Foken and Wichura 1996; Deshmukh and others 2014). Methane fluxes were also filtered to exclude periods where the received signal strength indicator (RSSI) for the LI-7700 was less than 30% (Podgrajsek and others 2014), and periods where it was determined that greater than 10% of the flux footprint were from outside the lake. The minimum wind speed filter of 1 m s^−1^ used by Podgrajsek and others (2014) was not applicable to this study because of the lower measurement height.

### Physicochemical Parameters and Diffusive Emission Rates

Unfiltered water samples were collected from a depth of 0.1 m at six sites (Figure 1; EOF, EFL, EMB, ECP, ENN, and EUS) every 3 weeks and analyzed for total phosphorus (TP), total nitrogen (TN), and chlorophyll *a*. Automated colorimetry (Lachat Instruments QuickChem 8000 Flow Injection Autoanalyzer, Loveland, CO, USA) was used to measure TP (Prokopy 1992) and TN (American Public Health Association 1995, followed by Wendt 1995) following acid persulfate and alkaline persulfate digestion, respectively. Chlorophyll samples were filtered (0.45 µm pore size) and the filtrate extracted with acetone. The absorbance of the extract was measured at 664, 647, and 630 nm with a spectrophotometer, and the chlorophyll *a* concentration was calculated using the trichromatic method (American Public Health Association 2012).

Dissolved oxygen and water temperature were measured at 1.5 m depth intervals at the deepest site (EOF) every two weeks using a data sonde (YSI, Yellow Springs, OH). Dissolved oxygen and water temperature were also measured at a depth 0.1 m below the water surface and 1 m above the sediment–water interface each time a trap was sampled.

A sample for dissolved CH_4_ analysis was collected from a depth of 0.1 m during all trap visits from July 1 through December 11, 2015, with the exception of the traps sampled during the week of the experimental drawdown. Dissolved CH_4_ samples were also collected at 1 m depth intervals at the deepest site (EOF) on four dates (July 8, August 26, September 30, and December 12, 2015). Dissolved CH_4_ was sampled via headspace equilibration after collecting a 120-ml water sample using a 140-ml plastic syringe equipped with a 2-way stopcock. Immediately after the sample was collected, 20 ml of ultra-high-purity helium was added to the syringe that was then shaken for 5 min, and the headspace gas transferred to a pre-evacuated 12-ml screw-top soda glass vial capped with a silicone-coated Teflon septa stacked on top of a chlorobutyl septa (Labco Ltd, UK). The CH_4_ partial pressure in the gas sample was measured via gas chromatography (see above), and the CH_4_ concentration in the water was calculated from a mass balance of the liquid and gas in the syringe following:

3 $$ ({\text{CH}}_{{ 4 , {\text{liq}}}}^{0} )({\text{V}}_{\text{liq}} ) = ({\text{CH}}_{{ 4 , {\text{liq}}}} )({\text{V}}_{\text{liq}} ) + ({\text{CH}}_{{ 4 , {\text{gas}}}} )({\text{V}}_{\text{gas}} ) $$

where CH_4_,_liq_^0^ is the CH_4_ concentration (µmol l^−1^) in the original water sample, *V*_liq_ and *V*_gas_ are the volumes (l) of liquid and gas in the syringe, and CH_4_,_liq_ and CH_4_,_gas_ are the CH_4_ concentrations (µmol l^−1^) in the liquid and gas, respectively, after the headspace equilibration. CH_4_,_liq_ was calculated from CH_4_,_gas_, the CH_4_ Bunsen solubility coefficient for the temperature of the headspace equilibration, and the pressure of the headspace equilibration, which was assumed to be equal to that of the atmosphere. Atmospheric pressure was monitored continuously at the EFL sampling station (Solinst).

The equilibrium-dissolved CH_4_ concentration in the reservoir (CH_4_,_eq_; µmol l^−1^) at the time of the dissolved gas sampling is calculated as:

4 $$ {\text{CH}}_{{ 4 , {\text{eq}}}} = ({\text{CH}}_{{ 4 , {\text{Atm}}}} )(\beta_{\text{T}} )(P_{\text{Atm}} ) $$

where CH_4_,_Atm_ is the global average CH_4_ partial pressure in the atmosphere (1.85 ppm, http://www.esrl.noaa.gov/gmd/ccgg/trends_CH4/), *β*_T_ is the Bunsen solubility coefficient for CH_4_ at the temperature of the water, and *P*_Atm_ is the atmospheric pressure (atm).

Diffusive CH_4_ flux (mg CH_4_ m^−2^ day^−1^) is calculated according to:

5 $$ {\text{Diffusive}}\;{\text{CH}}_{ 4} \;{\text{flux}} = k_{{{\text{CH}}_{ 4} }} ({\text{CH}}_{{ 4 , {\text{liq}}}}^{0} - {\text{CH}}_{{ 4 , {\text{eq}}}} )0.16 $$

where *k*_CH4_ (cm h^−1^) is the CH_4_ gas transfer velocity (Wanninkhof and others 2009) and 0.16 is a unit conversion constant. *k*_CH4_ was assumed to be equal to the mean *k*_CH4_ measured in a survey of 115 sites in Harsha Lake (7.3 ± 0.9 cm h^−1^) in 2014 (Beaulieu and others 2016).

### Total Flux Scaled to Lake

A previous survey of CH_4_ fluxes from 115 sites in Harsha Lake demonstrated that the river–reservoir transition zone is the source of 41% of total CH_4_ emissions from the reservoir (Beaulieu and others 2016). We used this scaling factor to generate a system-scale estimate of the CH_4_ flux rate from the measurements made at the nine monitoring stations following:

6 $$ {\text{System}}\;{\text{scale}}\;{\text{CH}}_{ 4} \;{\text{flux}} = [{\text{tributary}}\;{\text{flux}} \times 0.41] + [{\text{open}}\;{\text{water}}\;{\text{flux}} \times 0.59] $$

where the tributary flux is the mean flux measured at EUS, EUS2, and EUS3. Open water flux is the mean flux measured at the remaining sites.

### Statistical Analysis

The effect of water-level change on the ebullitive CH_4_ flux was determined separately for the experimental drawdown and periods outside of the drawdown. The effect of the experimental drawdown was assessed by comparing the ebullitive CH_4_ fluxes immediately prior to the experiment to those measured during the experiment. The effect of changes in water level and barometric pressure outside of the experimental drawdown was assessed using linear models. The dependent variable was the ebullitive CH_4_ flux during each deployment at each station (*n* = 254), and the independent variable was the change in water level or barometric pressure during each deployment. The linear models also include ‘site’ as a main effect and the interaction of site and ‘atmospheric pressure change’ or ‘water-level change.’

**Figure 2:**
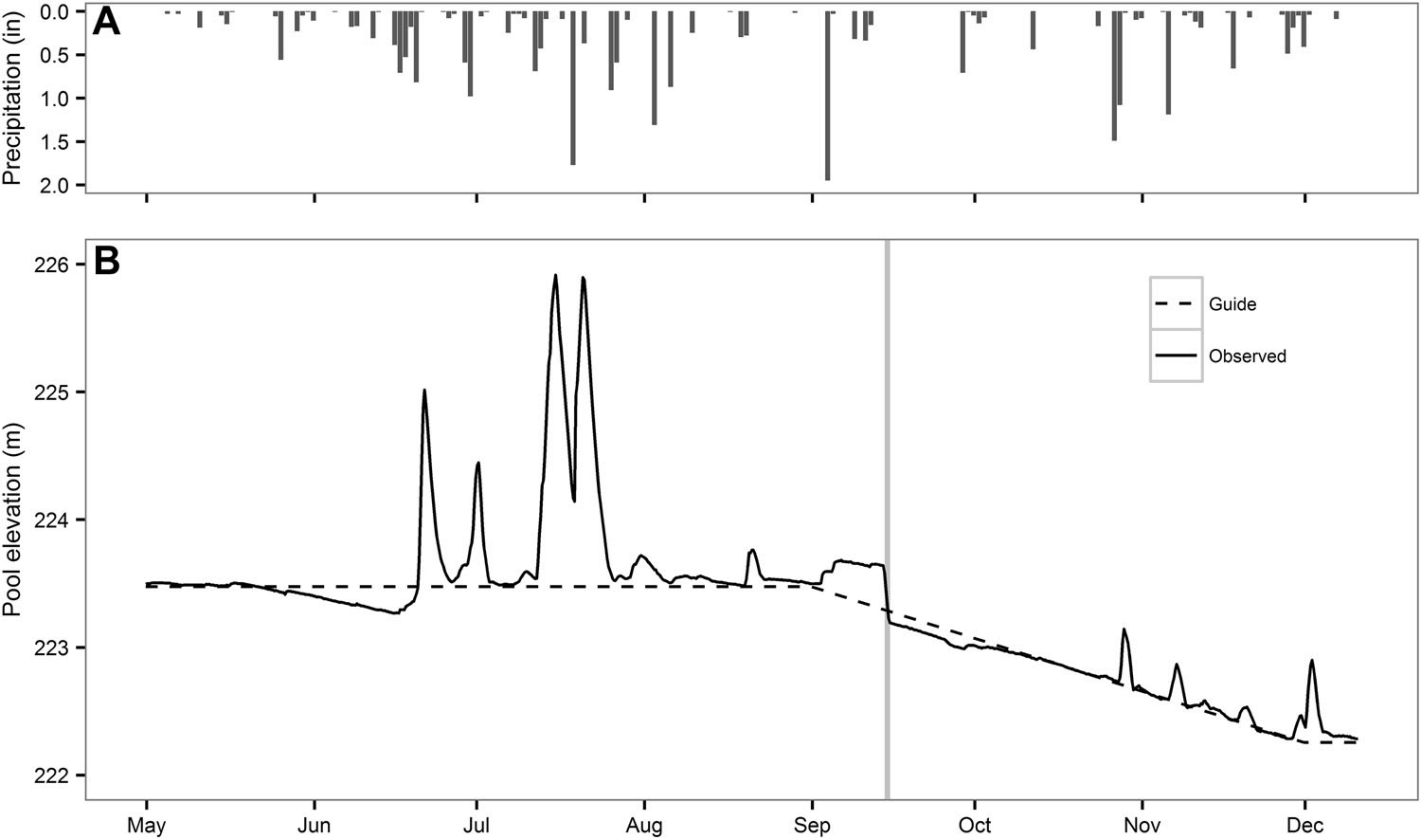
**A** Precipitation and **B** water levels at William H. Harsha Lake from May through December 2015. The *dashed black line* in panel B represents the management target and the *black line* represents the observed water levels. The *vertical gray line* represents the duration of the experimental water-level drawdown (Color figure online).

The emptying of the pool following large precipitation events in June and July led to several periods when the rate of water-level decline was similar to that during the experimental drawdown (Figure 2B). To determine whether these falling water levels increased the ebullitive CH_4_ flux, we compared the ebullitive CH_4_ flux during these periods of water-level declines to those measured immediately prior to the decline. All statistical analyses were performed using R (R Development Core Team 2016).

## Results

### Physical and Biogeochemical Setting

Harsha Lake was thermally stratified from late May, when the first depth profile was collected, through mid-October (Figure S2). Dissolved oxygen was less than 0.1 mg l^−1^ in the hypolimnion during the period of stratification and less than 3 mg l^−1^ throughout the lake during fall turnover. Dissolved CH_4_ concentration in the hypolimnion progressively increased during the period of thermal stratification, exceeding 1000 µmol l^−1^ by the end of September (Figure S3).

Mean TN and TP at a depth of 0.1 m were 1370 and 144 µg l^−1^, respectively, across sites and dates (Table 1). Chlorophyll *a* was low in April (mean = 9 µg l^−1^), high during the warm months (June–September mean = 50.1 µg l^−1^) and returned to low levels in the fall (October–December mean = 5.9 µg l^−1^). Dissolved CH_4_ was supersaturated in the surface waters at all sites and sampling dates. Dissolved CH_4_ at a depth of 0.1 m ranged from 0.03 to 70.2 µmol l^−1^ with an overall mean of 1.51 µmol l^−1^. On average, the surface waters were supersaturated with CH_4_ by a factor of 544 relative to the mean equilibrium concentration of 0.0027 µmol l^−1^.

**Table 1:** Monthly Mean (SE) Total Nitrogen (TN; µg N l^−1^), Total Phosphorus (TP; µg P l^−1^), Chlorophyll *a* (Chl *a*; µg l^−1^), Dissolved Methane (CH_4_; µmol l^−1^), and Dissolved Oxygen (DO; mg l^−1^) at the Monitoring Sites

	April	May	June	July	August	September	October	November	December
EOF									
TN	1385 (35)	998 (41)	1220 (112)	1630	1030	765 (53)	998	1125 (115)	1120
TP	261 (14)	97 (21)	95 (9)	106	82	84 (22)	137	93 (4)	91
Chl *a*	4 (1)	23 (2)	34 (19)	48	33	24 (1)	11	7 (2)	1
CH_4_	NA	NA	NA	0.5 (0.3)	0.2 (0)	0.4 (0.1)	0.3 (0.1)	0.1 (0.04)	0.1
DO	NA	NA	NA	15.7 (2.04)	8.48 (0.56)	11.1 (1.1)	4.21 (1.58)	4.46 (0.13)	6.85
EMB									
TN	1686 (227)	1170 (116)	1489 (58)	1467 (133)	1047 (124)	873 (78)	1447 (279)	1300 (223)	1581 (395)
TP	382 (93)	163 (46)	85 (14)	129 (34)	118 (47)	113 (33)	302 (103)	177 (85)	217 (87)
Chl *a*	8 (1)	21 (4)	66 (5)	60 (5)	41 (3)	24 (3)	7 (2)	5 (1)	1 (0)
CH_4_	NA	NA	NA	0.6 (0.3)	0.3 (0.1)	0.3 (0.1)	0.4 (0.1)	0.09 (0.03)	0.05
DO	NA	NA	NA	14.97 (1.65)	8.37 (0.92)	8.76 (2.17)	4.62 (0.86)	5.06 (0.27)	6.78 (0.75)
EFL									
TN	2193 (70)	1417 (145)	1591 (74)	1500 (121)	1017 (141)	916 (94)	1810	1923 (33)	1913
TP	587 (14)	222 (67)	124 (23)	126 (34)	122 (59)	139 (52)	480	426 (20)	436
Chl *a*	5 (1)	22 (5)	70 (8)	60 (3)	34 (3)	30 (4)	12	6 (3)	1
CH_4_	NA	NA	NA	0.4 (0.1)	0.3 (0)	3.1 (2.8)	0.5 (0.2)	0.14 (0.03)	0.04
DO	NA	NA	NA	14.93 (1.31)	7.31 (0.91)	7.08 (2.65)	4.84 (0.96)	5.81 (0.31)	5.95
EEB									
CH_4_	NA	NA	NA	1.1 (0.8)	0.5 (0.1)	0.6 (0.1)	0.4 (0.1)	0.14 (0.05)	0.6 (0.2)
DO	NA	NA	17.61	11.6 (1.43)	9.85 (1.07)	9.88 (2.04)	4.43 (0.85)	5.8 (0.21)	7.18 (0.01)
ECP									
TN	1258 (12)	828 (12)	1873 (315)	1280	1002	781 (85)	957	1139 (26)	1195
TP	249 (14)	69 (6)	163 (28)	170	107	74 (3)	128	93 (7)	98
Chl *a*	8 (7)	13 (1)	34 (10)	118	47	32 (11)	12	6 (1)	1
CH_4_	NA	NA	NA	0.4 (0.2)	0.5 (0.1)	0.3 (0)	0.4 (0.2)	0.11 (0.06)	0.4 (0.1)
DO	NA	NA	NA	10.93 (1.18)	9.76 (1.13)	6.96	4.81 (0.68)	5.66 (0.41)	7.01 (0.15)
ENN									
TN	1315 (45)	1035 (46)	1716 (109)	1388 (195)	918 (47)	827 (36)	949	1125 (85)	1220
TP	249 (23)	92 (9)	106 (11)	141 (11)	97 (7)	81 (6)	125	97 (10)	101
Chl *a*	11 (3)	28 (10)	80 (6)	53 (2)	48 (4)	38 (5)	12	6 (2)	1
CH_4_	NA	NA	NA	0.6 (0.4)	0.6 (0.2)	0.8 (0.3)	0.4 (0.1)	0.2 (0.1)	0.4 (0.2)
DO	NA	NA	NA	11.02 (0.94)	10.8 (1.29)	9.96 (2.82)	5.29 (0.44)	6.21 (0.26)	7.06 (0.12)
EUS2									
CH_4_	NA	NA	NA	3.2 (2.3)	1.5 (0.1)	2.3 (0)	19.4 (16.9)	3.1 (0.5)	3.8 (3.1)
DO	NA	NA	NA	9.85 (1.31)	10.13 (1.05)	4.41 (2.71)	6.5 (0.53)	6.71 (0.56)	9.29 (0.31)
EUS									
TN	1480 (210)	1260 (0)	2837 (490)	1200	1150	1268 (342)	1290	1295 (45)	1490
TP	255 (23)	182 (15)	264 (28)	165	166	199 (67)	192	131 (24)	168
Chl *a*	16 (4)	52 (1)	92 (26)	66	59	62 (38)	29	5 (0)	1
CH_4_	NA	NA	NA	2.4 (1.7)	1.3 (0.4)	1.5 (0.2)	1.8 (0.3)	3.1 (0.8)	4.1 (3.3)
DO	NA	NA	NA	8.36 (0.99)	9.43 (0.44)	8.46 (0.12)	6.43 (0.52)	6.66 (0.54)	8.56 (1.1)
EUS3									
CH_4_	NA	NA	NA	2.8 (2.3)	1.8 (0.5)	1.6 (0)	2 (0.6)	3.7 (0.9)	3.5 (2.8)
DO	NA	NA	16.09	9.35 (2.19)	9.93 (0.88)	8.87 (0.73)	6.56 (0.75)	6.84 (0.59)	9.65 (0.03)

*Samples were collected from a depth of 0.1* *m. NA indicates data not available. No standard error provided if only one sample was collected in a month.*

Below average precipitation in May and early June (Figure 2A) caused the pool elevation to fall 0.3 m below the management target (Figure 2B), but several large precipitation events in late June and July caused water levels to rise, reaching a maximum value 2.4 m above the management target. The pool elevation largely followed the guide curve in August, but strongly deviated from the guide curve during the experimental treatment in the first half of September. On September 5, 9 days prior to the planned drawdown experiment, the watershed received 1.95 inches of rain causing the pool elevation to rise by 0.17 m. This pool elevation was maintained until 9:00 am on September 14, at which point 0.46 m of water was spilled off the pool in 24 h. After the experimental treatment, the pool elevation fell at a rate closely approximating the guide curve.

### Ebullitive and Diffusive Flux

There were 254 individual automated trap deployments during the study with a median deployment duration of 6.97 days. In 69% of the deployments, the differential pressure sensor reached its maximum value and stopped collecting useful data before the trap was sampled. The median period of time over which the pressure sensor collected useful data (that is, integration period) was 14 h for the three traps with a 2.5-cm-diameter collection chamber deployed in the river–reservoir transition zone (EUS, EUS2, and EUS3). The median integration period for all other automated traps was 41 h.

Uncertainty in the circuit calibration and the magnitude of the electronic noise are propagated through equation 2 to estimate uncertainty in the ebullitive CH_4_ flux. All circuit calibrations had an *r* ^2^ of at least 0.994, and the mean standard deviation among pre- and post-deployment calibrations was 0.0635 cm V^−1^, equivalent to 2.1% of the mean calibration coefficient (30.41 cm V^−1^). The mean electronic noise from long-term deployments under constant differential pressure was 0.0005 V. These error terms resulted in an uncertainty in the ebullitive CH_4_ flux of 8.2%, on average.

**Figure 3:**
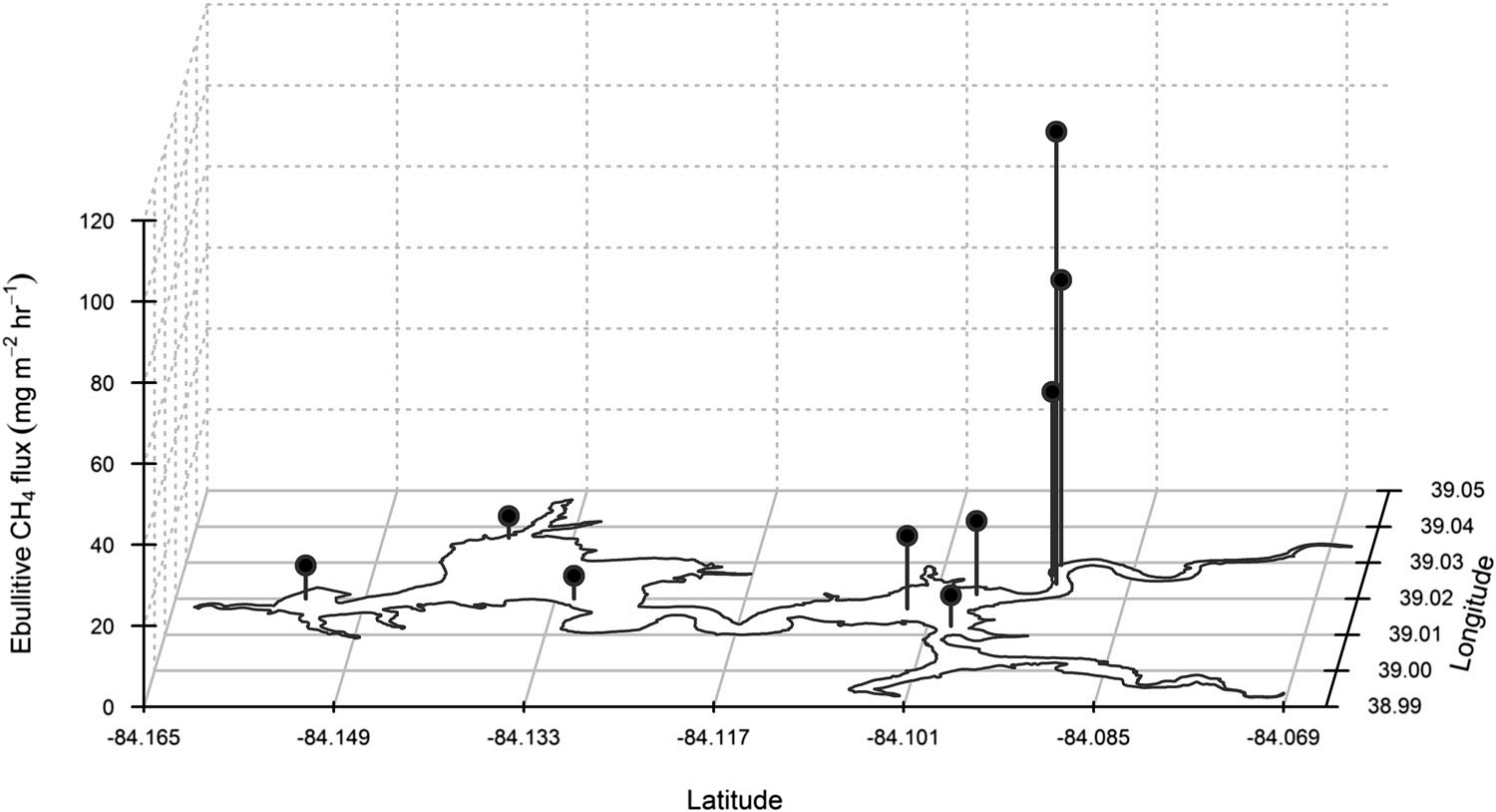
Mean ebullitive methane (CH_4_) flux rate measured at the nine monitoring stations during the study. The reservoir perimeter is indicated in *black* on the *xy* plane, and the magnitude of the flux is indicated by the height of the *black line* according the scale on the *z*-axis.

**Figure 4:**
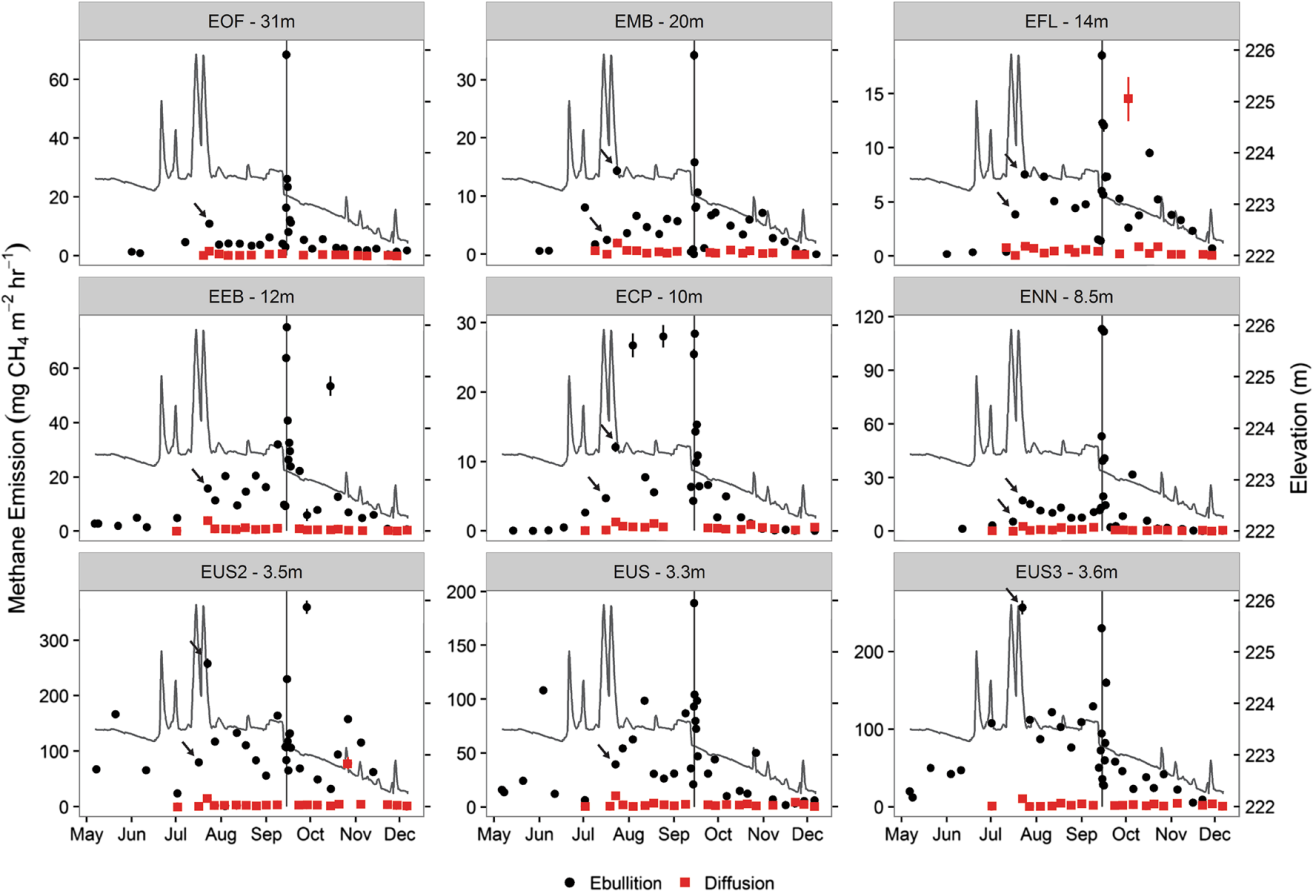
Ebullitive and diffusive methane (CH_4_) flux time series at each of the nine monitoring sites. The name and depth of the sampling site are shown in the *gray header* at the *top* of each panel. *Error bars* represent the 95% confidence interval. The *vertical gray line* in each panel represents the duration of the experimental drawdown, and the *dark gray line* indicates the pool elevation. *Arrows* highlight observations made when water levels were falling due to the release of stored storm water.

Methane partial pressure in collected bubbles ranged from 15.6 to 91.7% (mean = 64%) and was greater in the three sites located near the river inlet (EUS, EUS2, EUS3; mean = 72.4%) than the other sites (mean = 58.8%) (Table 2). Mean ebullitive CH_4_ fluxes across the study (that is, site means across the 7-month observation period, excluding drawdown experiment) ranged from 4.0 to 109.7 mg CH_4_ m^−2^ h^−1^ were highest in the river–reservoir transition zone (EUS, EUS2, ESU3) and lowest in the deeper waters in the west basin (Figure 3). The mean system-scale ebullitive CH_4_ flux during the study, excluding the drawdown experiment, was 32.3 mg CH_4_ m^−2^ h^−1^. The mean diffusive CH_4_ flux ranged from 0.39 to 6.75 mg CH_4_ m^−2^ h^−1^ across the nine monitoring sites, with the highest rates occurring in the river–reservoir transition zone (Figure 4). At the system scale, diffusive emissions (mean = 2.0 mg CH_4_ m^−2^ h^−1^) comprised 6% of the total mean flux (ebullitive + diffusive; mean = 34.3 mg CH_4_ m^−2^ h^−1^).

**Table 2:** Mean (SD) Methane (CH_4_) Content of Collected Bubbles During the Study

	EOF	EMB	EFL	EEB	ECP	ENN	EUS	EUS2	EUS3
CH_4_ (%)	62.5 (8.2)	60.9 (12.1)	62.9 (11.4)	52.9 (17.3)	54.9 (15.0)	59.2 (18.5)	69.0 (18.5)	75.2 (11.8)	72.9 (11.8)

Ebullitive CH_4_ fluxes increased by a factor of 1.4–77 during the experimental water-level drawdown, but returned to background levels within 1 week (Figure 4). Diffusive emission rates were not measured during the drawdown experiment.

### Correlation with Other Forcing Factors

When observations from the drawdown experiment were excluded, ebullitive CH_4_ flux was unrelated to changes in barometric pressure (*p* = 0.58), but increased with falling water levels (*p* = 0.006). The effect of water-level change varied by site (significant site*water-level change interaction, *p* < 0.001) was strongest in the three shallowest sites (EUS, EUS2, and EUS3) and had the greatest effect at EUS3 (increase of 71 mg CH_4_ m^−2^ h^−1^ per 1 m decline in water level at EUS3).

### Eddy Flux Results

**Figure 5:**
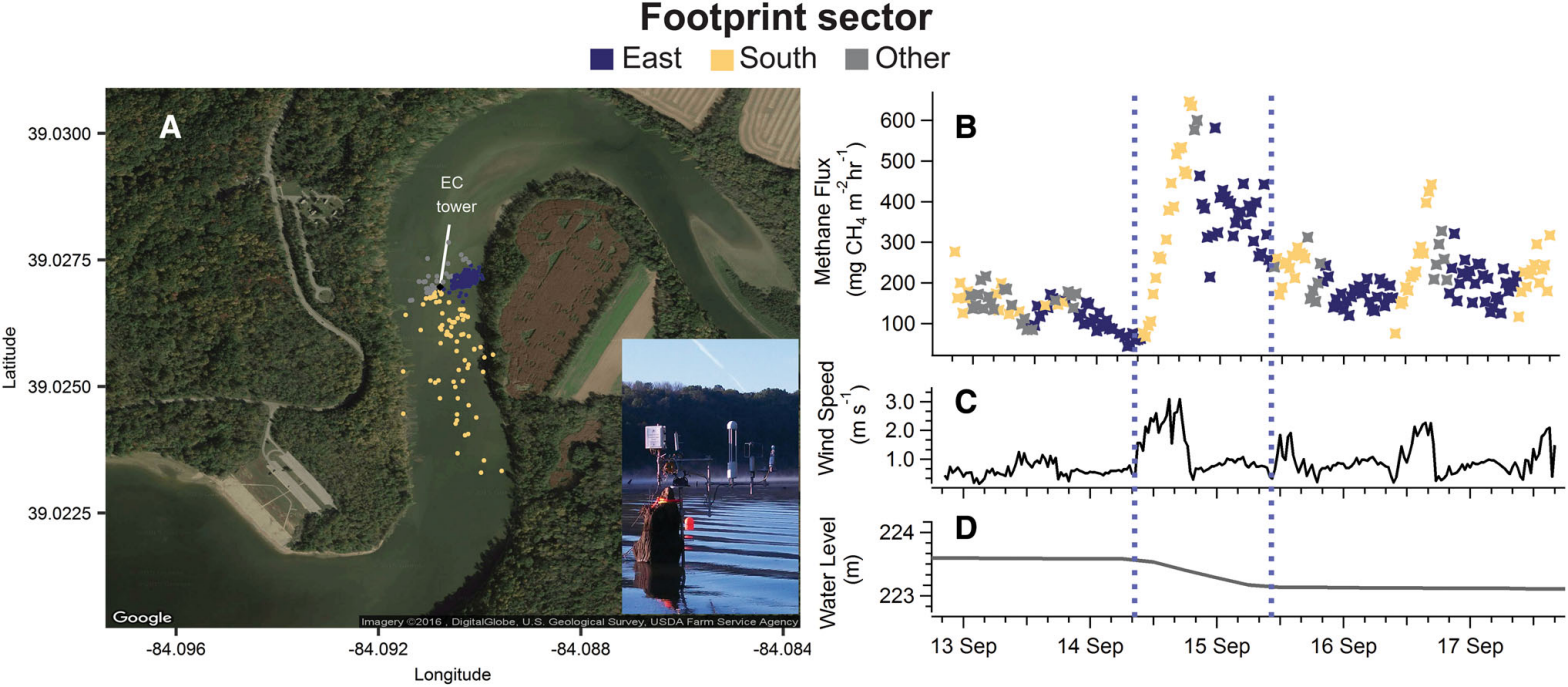
**A** The eddy covariance (EC) measurement footprint that includes 90% of the contributing area for each half-hour averaging period. The distance and angle between a point and the flux tower indicate the maximum extent and orientation, respectively, of the footprint during one half-hour averaging period. EC footprint areas extending to the south, east, and other directions are indicated by *yellow*, *blue*, and *gray dots*, respectively. The* inset* image is the flux tower deployed over the water surface. **B** Methane (CH_4_) flux, **C** wind speed, and **D** water-level time series during the EC tower deployment. The *blue dashed lines* indicate the start and end of the experimental drawdown. The *color* of the markers in **B** corresponds to the orientation of the EC footprint area (see panel A) (Color figure online).

The EC measurement footprint is a function of sensor height, atmospheric stability, surface roughness, and wind direction (Kljun and others 2004). Both wind speed and direction displayed a strong diurnal pattern during the drawdown period. Low-speed winds (mean = 0.7 m s^−1^) from the east by northeast direction were prevalent at night (8:00 pm–8:00 am), whereas moderate-wind speeds (mean = 1.3 m s^−1^) from the south were prevalent during the day (9:00 am–5:00 pm) (Figure S4A). This resulted in EC measurement footprints that extended an average of 142 m to the south during the day and 45 m to the east by northeast at night (Figure 5A). The EC measurement footprint that captured 90% of the contributing area extended over land in only five of the 232 half-hour CH_4_ flux estimates, and these periods were filtered from the final dataset. Applying this filter along with the other quality control checks outlined in the methods resulted in acceptance of 94.8% of the 30-min CH_4_ flux data.

This acceptance rate is high relative to studies in terrestrial systems, but similar to the acceptance rates reported in other reservoir studies (Liu and others 2012, 2016). Over land, an important contributor to lower acceptance rates is the filtering of stable nighttime periods that do not pass the test for developed turbulent conditions (Foken and Wichura 1996) or when the friction velocity (u_*_) is below a certain threshold (Barr and others 2013). In aquatic systems, heat transfer from the water surface to the overlying air at night leads to convective mixing and unstable atmospheric conditions. Although we did not measure water surface temperature in this study, both the sensible and latent heat fluxes (H and LE, respectively) were positive at night (Figure S4B, C), indicating heat transfer from the water surface to the air. The friction velocity was low at night (u_*_, Figure S4D), but since u_*_ is a measure of mechanically generated turbulence it is not a good indicator of stability regime over water bodies where buoyancy plays a larger role. Therefore, heat loss to the atmosphere at night and resulting buoyancy-generated turbulence lead to the overall high data acceptance rates in this study.

Prior to the drawdown (September 12, 8:30 pm–September 14, 8:30 am), the average (±SE) CH_4_ flux was 133 ± 5.4 mg CH_4_ m^−2^ h^−1^ (Figure 5B). The average CH_4_ flux during the 24-h experimental drawdown (September 14, 9:30 am–September 15, 9:00 am) was 370 ± 20 mg CH_4_ m^−2^ h^−1^, with a maximum emission rate of 646 mg CH_4_ m^−2^ h^−1^ observed on September 14 at 6:00 pm. The average CH_4_ flux after the experimental drawdown (September 15, 9:30 am–September 17, 4:00 pm) was 212 ± 5.9 mg CH_4_ m^−2^ h^−1^.

## Discussion

**Figure 6:**
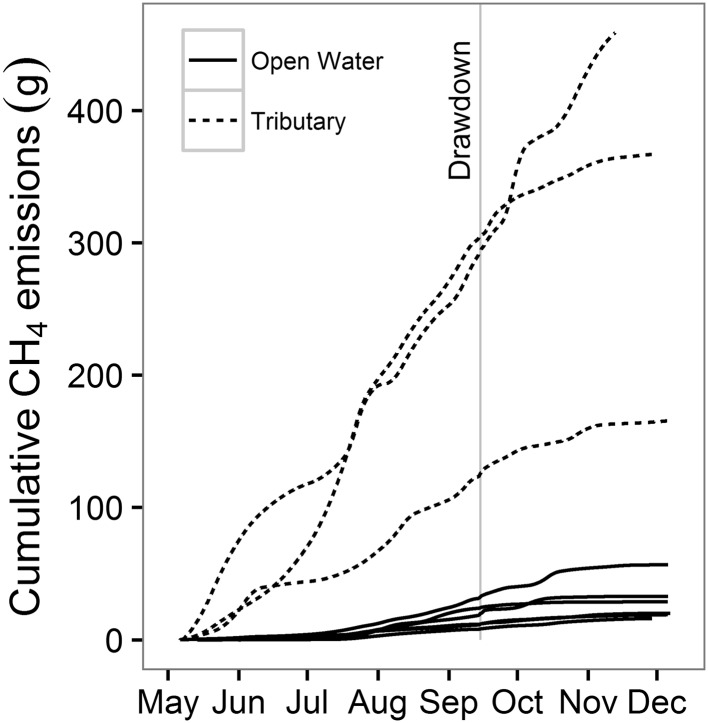
Cumulative methane (CH_4_) emissions from the nine monitoring stations from May through December 2015. The *vertical gray line* represents the duration of the 24-h drawdown experiment.

Methane fluxes from Harsha Lake were approximately tenfold greater in the river–reservoir transition zone (mean = 72.8 mg CH_4_ m^−2^ h^−1^, excluding drawdown) than in the main body of the reservoir (mean = 7.6 mg CH_4_ m^−2^ h^−1^, excluding drawdown) (Figures 3, 6). This is consistent with previous surveys in Harsha Lake, which have shown that the river–reservoir transition zone is a disproportionately important CH_4_ source in the system (Beaulieu and others 2014, 2016). This spatial pattern has been documented for a wide variety of reservoirs including subtropical systems in Australia (Grinham and others 2011; Musenze and others 2014; Sturm and others 2014), reservoirs in the Pacific Northwest of the USA (Harrison and others 2017), and a large tropical reservoir (5400 km^2^ surface area) in Africa (DelSontro and others 2011). River–reservoir transition zones are characterized by decreasing water velocities and the deposition of suspended sediment (Thornton 1990). When the sedimentation rate exceeds the carbon mineralization rate, labile carbon can be buried into deeper sediments where anoxia and methanogenesis are prevalent, thereby stimulating CH_4_ production (Sobek and others 2009; Maeck and others 2013). This mechanism, combined with shallow water depths and low hydrostatic pressure, may account for the persistently high ebullitive CH_4_ flux below the tributary inputs.

The mean system-scale total CH_4_ flux (ebullition + diffusion) during the study (34.3 mg CH_4_ m^−2^ h^−1^) is higher than previously reported for Harsha Lake during the warm weather months (6.5–8.3 mg CH_4_ m^−2^ h^−1^; Beaulieu and others 2014, 2016), possibly due to methodological differences among studies. The earlier studies measured CH_4_ fluxes (ebullitive and diffusive) using short-term (5–12 min) floating chamber deployments, whereas the current work utilized continuous monitoring over hours to days. Short-term monitoring may miss rare, but large, ebullition events, thereby underestimating ebullition rates (Varadharajan and Hemond 2012; Maeck and others 2014). Furthermore, the flux rates measured using the EC tower in the current study are somewhat higher than those measured using inverted funnels at the three monitoring stations located within the EC tower footprint (Figures 5B, S5), possibly due to discrete hot spots affecting the tower and not the funnels, suggesting that the inverted funnel method is not overestimating the ebullitive CH_4_ flux in this study.

The mean system-scale total CH_4_ flux (ebullition + diffusion; 34.3 mg CH_4_ m^−2^ h^−1^) observed during this study is the highest yet reported for a reservoir (Figure S6), which contradicts earlier findings that high emission rates are restricted to tropical areas (Barros and others 2011). Our data join a growing body of the literature, indicating that temperate zone reservoirs in Europe and the USA can support CH_4_ emission rates as high as those reported for the tropics (DelSontro and others 2010; Gruca-Rokosz and others 2011; Maeck and others 2013; Harrison and others 2017).

The high CH_4_ fluxes from Harsha Lake may be linked to the high algal primary productivity of the reservoir. Methane emission rates have been shown to correlate with reservoir productivity at regional (West and others 2016; Harrison and others 2017) and global scales (Deemer and others 2016), suggesting that reservoir productivity may exert a strong control on CH_4_ emission rates. Algal productivity could directly enhance CH_4_ emissions by providing a source of labile carbon to methanogens. For example, laboratory studies have shown that additions of algal-derived carbon to aquatic sediments can stimulate CH_4_ production (West and others 2012). Algal productivity could also indirectly enhance CH_4_ emissions by promoting the development of anoxic conditions, which can stimulate methanogenesis and inhibit methanotrophy. Although CH_4_ emission rates were not related to dissolved oxygen (*p* = 0.17) in this study, CH_4_ concentrations in the anoxic hypolimnion exceeded 1000 µmol l^−1^ by late September (Figure S2), indicating substantial CH_4_ production and accumulation in anoxic waters.

### Emission Rate Response to Experimental Drawdown

The gas traps indicated that the experimental water-level drawdown at Harsha Lake triggered a synchronous system-wide increase in the ebullitive CH_4_ flux (Figure 4). At most sites, ebullitive CH_4_ flux during the drawdown was the highest recorded at any time during the 7-month monitoring period and exceeded fluxes observed during the 2–3 days immediately prior to the experiment at all sites. The CH_4_ flux rates measured with the EC tower were also higher during the drawdown, increasing by a factor of 5 relative to the average pre-drawdown flux rate. The EC flux data also indicated a strong diurnal cycle, with higher flux rates observed during the day than at night. This diurnal pattern in flux rates coincided with a diurnal pattern in wind speed, wind direction, and turbulent mixing, resulting in a southerly EC footprint during the day and an easterly footprint at night (Figure 5A). The diurnal pattern in flux rates could therefore be partly attributed to higher CH_4_ production rates in areas south of the EC tower than areas east of the tower, rather than a temporal pattern in CH_4_ flux. Nevertheless, flux rates from both the southerly-daytime and easterly-nighttime footprints were higher during the drawdown compared to the day before the drawdown, indicating that declining water levels triggered ebullition from both footprint areas.

**Figure 7:**
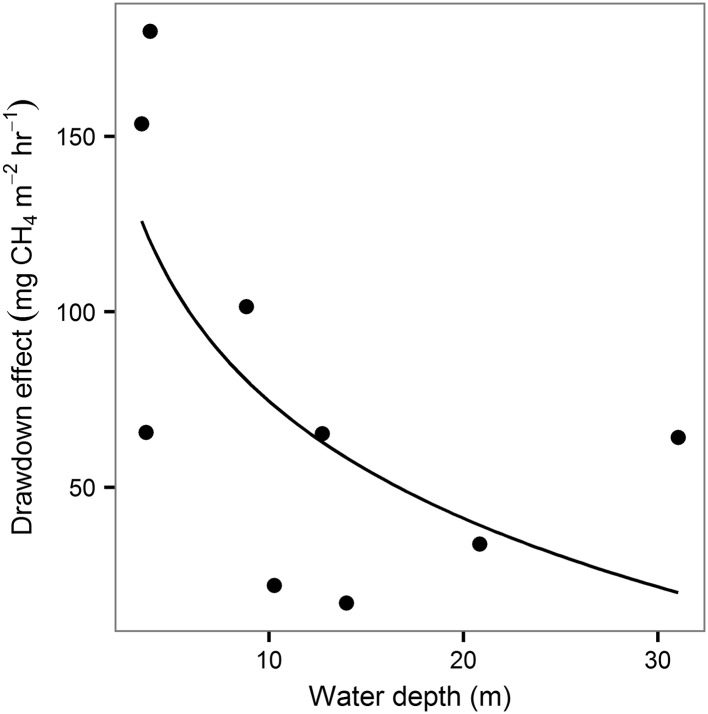
Relationship between the increase in the ebullitive methane (CH_4_) flux rate during the 24-h drawdown experiment and water depth. The drawdown effect is calculated as the difference between the maximum ebullitive CH_4_ flux during the experimental drawdown and that observed the day prior to the experiment.

The experiment clearly demonstrated that rapid decreases in water level can trigger ebullition in Harsha Lake, likely due to the release of sediment gas bubbles following the drop in hydrostatic pressure. Although ebullition rates were higher at all sites during the drawdown, the shallow sites responded most strongly (Figure 7). This pattern was also evident when the drawdown data were excluded from the analysis and is consistent with reports from a subtropical reservoir (Nam Theun 2, Southeast Asia) where the effect of water-level change was stronger in shallow than deep sites (Deshmukh and others 2014). This pattern may be partly explained by differences in the proportional change in hydrostatic pressure for a given change in water level among the sites. The 0.46 m drop in water level during the drawdown resulted in less than a 2% decrease in hydrostatic pressure at the sediment surface at the 30-m deep site compared to a 10% decrease at the shallow sites.

### Relationship with Water-Level Variation Outside of Drawdown

The rapid emptying of the pool following large precipitation events in June and July led to several periods when the rate of water-level decline was similar to that during the experimental drawdown. Ebullitive CH_4_ fluxes during these periods of water-level decline were on average fivefold greater than the preceding week (Figure 4). One potential explanation for this pattern is that the increase in hydrostatic pressure that accompanied the rising water levels allowed additional CH_4_ gas to accumulate in the sediments. This gas was subsequently released to the water column when water levels dropped and the system equilibrated to the lessened hydrostatic pressure.

Although rapidly falling water levels following precipitation events triggered elevated ebullitive CH_4_ fluxes, at most sites the magnitude of the response was only a fraction of that observed during the experimental drawdown, despite the fact that water levels dropped by as much as 2.4 m following storms, whereas water levels dropped by only 0.46 m during the experimental drawdown. One difference between these periods of water-level decline is the duration that the pool was held at the pre-spill water level. The volume of gas released from sediments during periods of water-level decline will be related to the difference between the amount of gas stored in the sediments prior to the drawdown and the storage capacity of the sediments after the drawdown. While storms caused water levels to rise significantly, the pool was maintained at these high levels for only a brief period, and it is possible that the duration was too short for microbial gas production to substantially increase the volume of gas stored in the sediment. Therefore, the difference between the pre-drawdown gas storage and post-drawdown gas holding capacity may not have been great during the post-storm drawdowns. By contrast, the experimental drawdown dropped the pool to the lowest level in 5 months. The pervasive ebullition observed at all monitoring stations prior to the drawdown indicates that the gas holding capacity of the sediments was fully saturated at this time. Therefore, the difference between the pre-drawdown gas storage and post-drawdown gas holding capacity of the sediment was likely greater during the experimental drawdown than the post-storm drawdowns, possibly explaining why the fall drawdown triggered a much stronger response in the ebullitive CH_4_ flux. The drawdown response may have been further enhanced by the 0.17 m rise in pool elevation that occurred nine days before the experiment. This change in pool elevation resulted from water that was stored in response to a 2 in storm event, rather than as a component of the experimental design. Nevertheless, the elevated hydrostatic pressure at the higher pool elevation likely allowed for additional gas to accumulate in the sediments, which was subsequently released to the water column during the drawdown.

Water levels continued to fall after the experimental drawdown, but at the much lower rate prescribed by the guide curve. Interestingly, there is little evidence in the data that the ebullitive CH_4_ flux was elevated during this period, possibly because the rate of water-level decline was too low to trigger a detectable increase in ebullition. It is also likely that falling water temperatures during the 2.5-month drawdown caused sediment CH_4_ production rates to decrease (Juutinen and others 2009; DelSontro and others 2010; Marotta and others 2014; Rasilo and others 2015), potentially offsetting the stimulatory effect of falling water levels on ebullition.

### Cumulative Emissions

Although the ebullitive CH_4_ flux increased by as much as an order of magnitude during the experimental drawdown, these elevated emission rates were short lived and constituted only 3.3% of cumulative CH_4_ emissions from the sites during the study period (Figure 6). These results contrast markedly with results from Lacamas Lake, a eutrophic reservoir located in the Pacific Northwest USA where CH_4_ emissions during an annual water-level drawdown constituted more than 90% of annual CH_4_ emissions (Harrison and others 2017). Annual drawdowns in Lacamas Lake were deeper (1.5–2 m) and sustained for longer periods (1–2 weeks) than the experimental drawdown in this study, both factors which may help explain the divergent results between these two studies. Importantly, background ebullitive CH_4_ flux rates differ markedly between Harsha and Lacamas lakes. In Lacamas, where ebullition rates were measured at multiple sites including the river–reservoir transition zone, rates outside of the drawdown periods were generally quite low, whereas the background ebullitive CH_4_ flux rate at Harsha Lake is among the highest reported for any reservoir (Figure S6). Given the high ebullitive CH_4_ flux rates at Harsha Lake throughout the warm weather season, short-term increases during periods of rapidly falling water levels do not greatly increase annual emissions from the reservoir.

Although the one-time short-term period of rapidly falling water levels did not greatly increase annual CH_4_ emissions from Harsha Lake, the effect of multiple periods of rapidly falling water levels should be considered. While this scenario is rare at Harsha Lake, it is the norm at nearby C. J. Brown reservoir where the fall drawdown consists of rapid water-level declines during the weekends to provide recreational flows to a downstream whitewater park (http://www.lrl-wc.usace.army.mil/plots/cbr.htm). Similarly, hydropower reservoirs managed for ‘hydropeaking,’ a process whereby river flows are increased during the day when energy demand is high and decreased at night when demand is low (Førsund 2007), are subject to regular periods of rapid water-level decline. Additional research is required to assess the impact of multiple drawdown events on annual CH_4_ emissions.

### Management Implications

Although this study did not test management strategies for reducing reservoir-CH_4_ emissions, it adds to the growing body of evidence that water-level drawdowns can stimulate ebullitive CH_4_ flux in reservoirs (Deshmukh and others 2014; Harrison and others 2017), thereby establishing a connection between water-level management and CH_4_ emissions. Changes to water-level drawdown procedures could reduce annual scale emissions if the changes increase the fraction of CH_4_ that is oxidized by methanotrophs (CH_4_-oxidizing bacteria) to CO_2_, a much less potent greenhouse gas (IPCC 2013). Aerobic CH_4_ oxidation at the metalimnion and anaerobic CH_4_ oxidation in the hypolimnion can be important CH_4_ sinks in stratified lakes and reservoirs (Rudd and Hamilton 1978; Fallon and others 1980; Guérin and Abril 2007; Sturm and others 2016). Methane oxidation can also be a substantial CH_4_ sink during fall overturn when oxygen-rich surface waters mix into deeper CH_4_-rich waters (Kankaala and others 2006, 2007). Overall, methanotrophs have been shown to oxidize up to 95% of the CH_4_ produced during the stratified period, substantially reducing CH_4_ emissions to the atmosphere (Rudd and Hamilton 1978; Fallon and others 1980; Guérin and Abril 2007; Bastviken and others 2008). However, the proportion of produced CH_4_ that is oxidized to CO_2_ can be greatly diminished in systems with active ebullition because rising bubbles rapidly pass through the water column and are largely unaffected by CH_4_ oxidation (Bastviken and others 2008), especially in shallow waters (McGinnis and others 2006). Water-level drawdowns that trigger ebullition may therefore increase the fraction of CH_4_ that escapes oxidation and is emitted to the atmosphere. In the absence of a drawdown, this CH_4_ may dissolve into sediment pore water, diffuse into the water column, and be oxidized to CO_2_. This may be particularly relevant in the fall, when water-level drawdowns are frequently conducted, because decreasing water temperature will enhance CH_4_ dissolution into porewaters. Furthermore, the temperature sensitivity of methanogens is much greater than that of methanotrophs (Borrel and others 2011) and falling water temperatures may cause the rate of CH_4_ oxidation to approach and even exceed the rate of CH_4_ production. The combined effects of falling water temperature on CH_4_ production, oxidation, and dissolution may cause a growing fraction of sediment CH_4_ gas to be oxidized to CO_2_ during the fall, assuming that the gas is not stripped from the sediment via drawdown-induced ebullition. Therefore, the management objective should be to retain CH_4_ gas within the sediments as long as possible in order to maximize CH_4_ oxidation. This might be accomplished by (1) delaying fall drawdowns until after the sediment CH_4_ gas load has been exhausted, or (2) conducting the drawdown at a rate of water-level decline that is too low to substantially enhance ebullition. Both of these approaches could be experimentally tested.

Taken together, the reservoir drawdown research conducted in the PNW (Harrison and others 2017) and this study suggest that altered water-level management has the greatest potential to mitigate CH_4_ emissions from reservoirs that are subject to frequent drawdown events or have background ebullition rates substantially lower than those in Harsha Lake. Recent work in unproductive reservoirs in the southeastern region of the USA indicates that these systems have low ebullitive CH_4_ fluxes during the summer months (0–0.7 mg CH_4_ m^−2^ h^−1^) and undergo water-level drawdown every fall (0.3–16 m) (Bevelhimer and others 2016). These systems meet the criteria for reservoirs where altered water-level management might mitigate emissions, and future research should determine whether emissions during the period of water-level drawdown constitute a disproportionate share of annual CH_4_ emissions.

Water-level management represents one approach for mitigating emissions from reservoirs. Another approach that may be effective for reservoirs with high background ebullition rates, such as Harsha Lake, is watershed nutrient management. Several studies have shown a correlation between CH_4_ emission rates and reservoir productivity (Deemer and others 2016; Harrison and others 2017), suggesting that watershed management strategies aimed at reducing nutrient loading to reservoirs may also reduce CH_4_ emissions. A careful consideration of the suite of management options for mitigating CH_4_ emissions from reservoirs will help minimize the greenhouse gas footprint of these important ecosystems.

## Electronic supplementary material

Below is the link to the electronic supplementary material.

Supplementary material 1 (DOCX 5753 kb)
