# Supplementary material for: Effects of an Experimental Water-level Drawdown on Methane Emissions from a Eutrophic Reservoir
Source: Ecosystems. 2017 Sep 5;21(4):657–74. doi: 10.1007/s10021-017-0176-2 (PMC6445499; doi:10.1007/s10021-017-0176-2)
Supplement: Supplementary file 1 — Supplementary material 1 (DOCX 5753 kb) [file 10021_2017_176_MOESM1_ESM.docx]

**SI Material for “Effects of an experimental water-level drawdown on methane emissions from a eutrophic reservoir”**

**Authors**

Jake J Beaulieu^1^, David A Balz^2^, M. Keith Birchfield^3^, John A Harrison^3^, Christopher T Nietch^1^, Michelle C Platz^1^, William C Squier^4^, Sarah Waldo^1^, John T Walker^5^, Karen M White^1^, Jade L Young^6^

^1^United States Environmental Protection Agency, Office of Research and Development, National Risk Management Research Laboratory, Cincinnati, Ohio, USA

^2^Pegasus Technical Services, Cincinnati, Ohio, USA

^3^Washington State University, School of the Environment, Vancouver, Washington, USA

^4^United States Environmental Protection Agency, National Enforcement Investigations Center, Denver, Colorado, USA

^5^United States Environmental Protection Agency, Office of Research and Development, National Risk Management Research Laboratory, Durham, North Carolina, USA

^6^United States Army Corps of Engineers, Louisville, Kentucky, USA


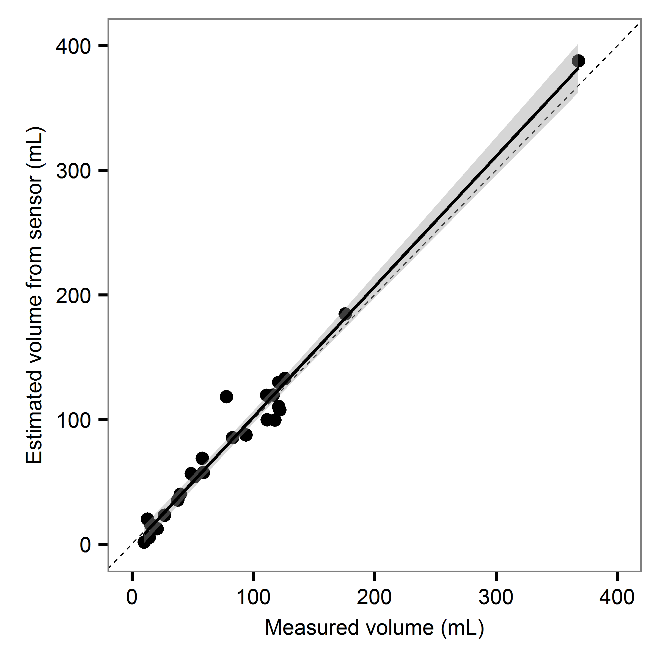


Fig. S1. Volume of gas in trap calculated from sensor versus volume of gas measured in trap. The solid line and shaded grey area represent the regression line and 95% confidence interval, respectively. The dashed grey line represents the 1:1 relationship. Figure only includes data from deployments where the differential pressure sensor was retrieved before the accumulated gas volume was great enough to saturate the sensor signal.


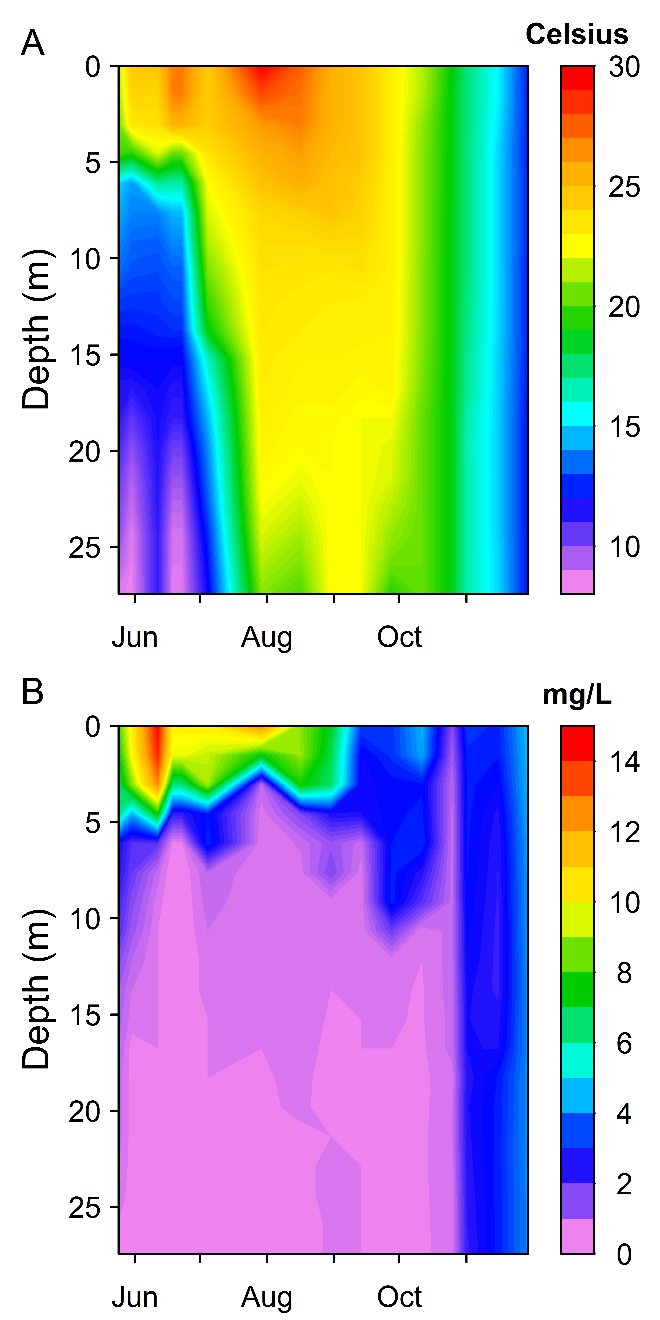


Fig. S2. Heat maps of A) water temperature and B) dissolved oxygen in Harsha Lake from May through December 2015.


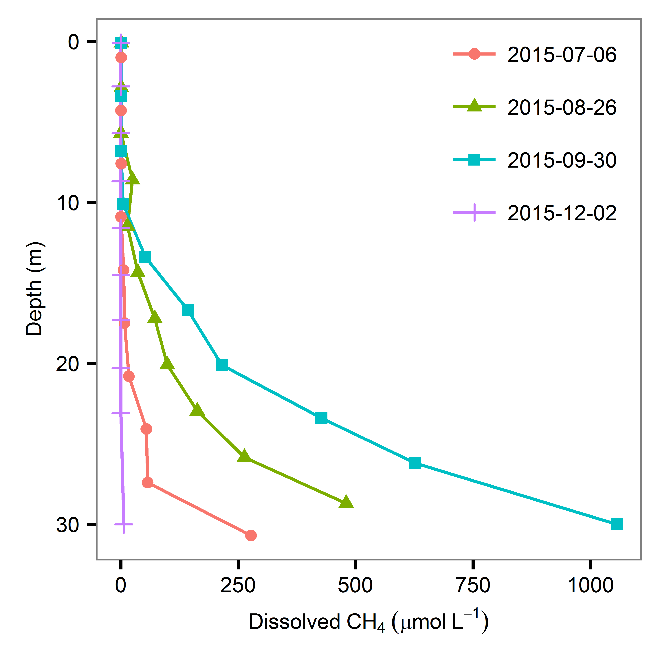


Fig. S3. Vertical profiles of dissolved CH_4_ concentration at the deepest monitoring site (EOF) on four dates.


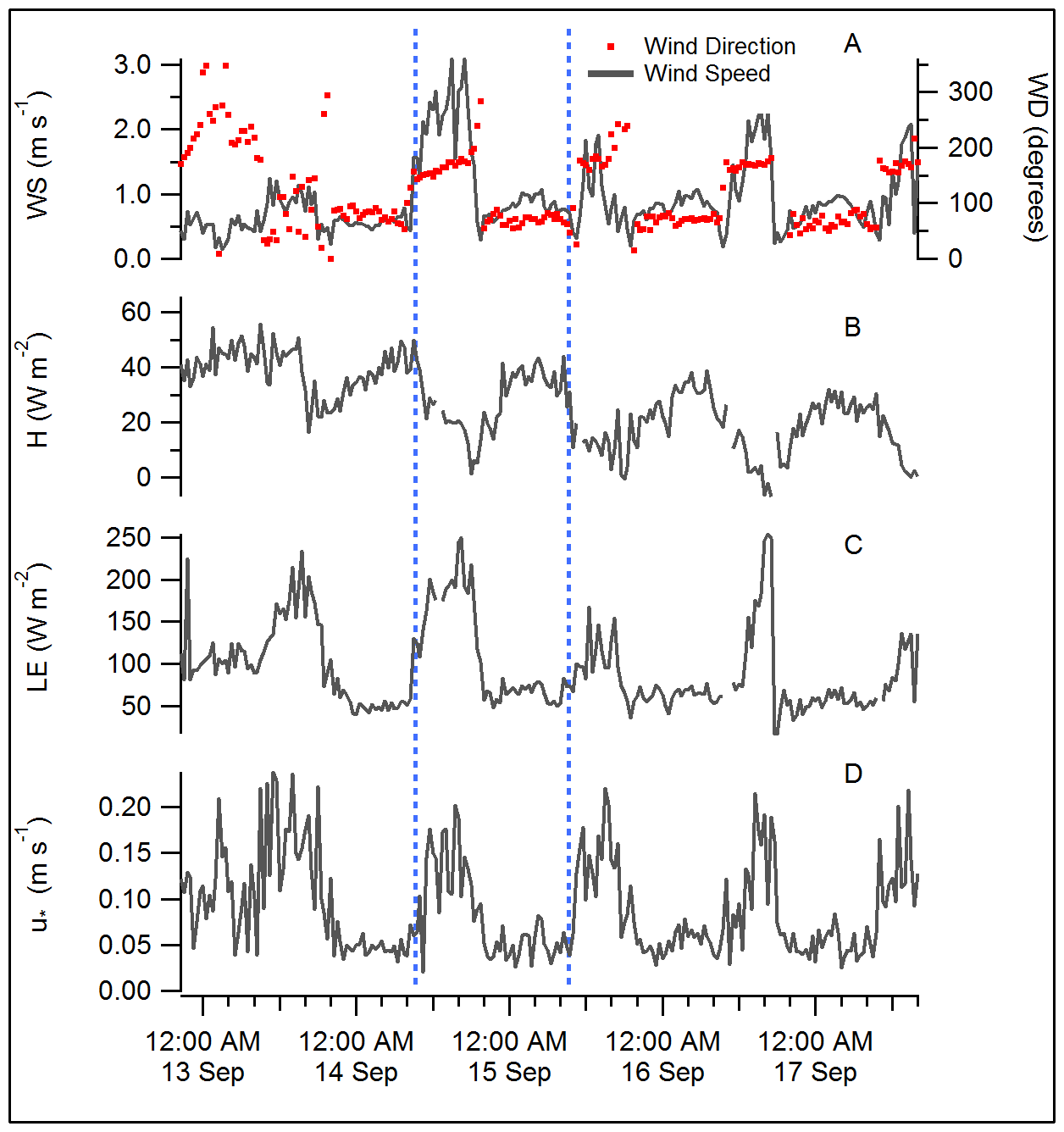


Fig. S4: Time series of 30-minute values for A) wind speed (WS, m s^-1^) and wind direction (WD, degrees), B) sensible heat flux (H, W m^-2^), C) latent heat flux (LE, W m^-2^), and D) friction velocity (u*, m s^-1^). The blue dashed lines indicate the start and end of the experimental drawdown.


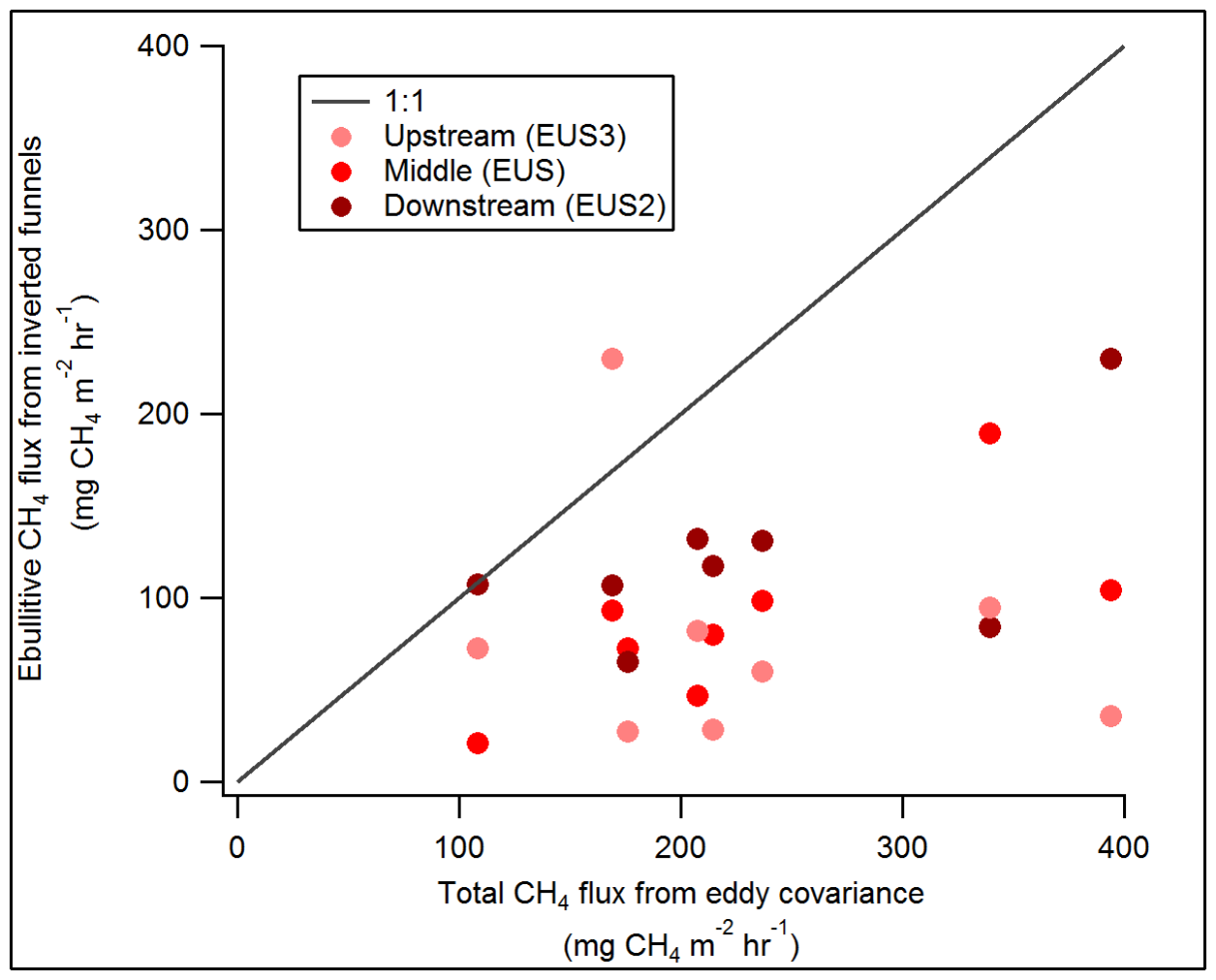


Fig. S5. Ebullitive methane (CH_4_) flux from inverted funnels located within the EC footprint versus total CH_4_ flux measured with the EC tower. Each point represents an emission rate estimate corresponding to the duration of the funnel deployment. The EC flux tower measurement represented by each point was calculated by integrating the total CH_4_ flux over each funnel deployment duration, then normalizing the cumulative emissions to the elapsed time.


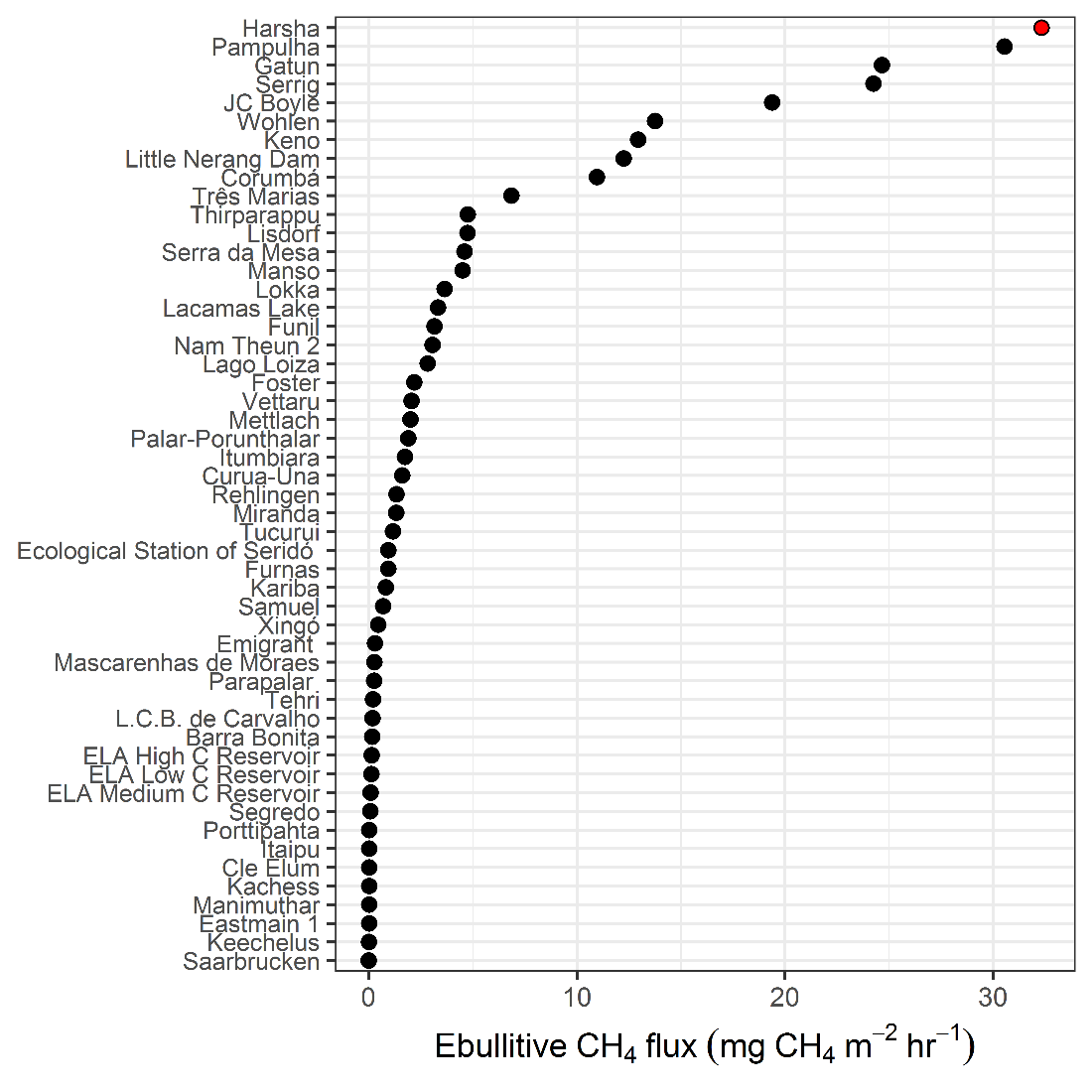


Fig. S6. Each dot represents a methane (CH_4_) ebullition rate reported in the literature for a reservoir. The red circle represents the system-scale mean for Harsha lake during the study. All other data were extracted from Deemer et al. ([2016](#_ENREF_1)).

References

Deemer, B. R., J. A. Harrison, S. Li, J. J. Beaulieu, T. DelSontro, N. Barros, J. F. Bezerra-Neto, S. M. Powers, M. A. dos Santos, and J. A. Vonk. 2016. Greenhouse Gas Emissions from Reservoir Water Surfaces: A New Global Synthesis. Bioscience, doi:10.1093/biosci/biw117
